# Evaluation of Legiolert for Quantification of *Legionella pneumophila* from Non-potable Water

**DOI:** 10.1007/s00284-018-1522-0

**Published:** 2018-07-06

**Authors:** Melanie M. Rech, Brian M. Swalla, Jason K. Dobranic

**Affiliations:** 1 EMSL Analytical, Inc., 1010 Yuma Street, Denver, CO 80204 USA; 2 IDEXX Laboratories, Inc., 1 Idexx Dr., Westbrook, ME 04092 USA; 3 EMSL Analytical, Inc., 5950 Fairbanks North Houston Rd, Houston, TX 77040 USA

## Abstract

Legiolert® is a new culture method for quantification of *Legionella pneumophila*, which is the primary species associated with Legionnaires’ disease. The test is based on a most probable number approach, and differs significantly from traditional culture methods by providing results at 7 days, rapid sample preparation and analysis, and objective interpretation of test results. In this study, we compared the performance of Legiolert with the U.S. Centers for Disease Control and Prevention (CDC) method for detection of *L. pneumophila* from non-potable samples, primarily comprising cooling tower waters. Our results demonstrated no significant difference between Legiolert and the CDC method for quantification of *L. pneumophila*. However, Legiolert showed a significant increase in sensitivity when water samples containing higher *L. pneumophila* concentrations were examined. Cooling tower waters often contain non-*Legionella* organisms (NLO) that interfere with traditional *Legionella* test methods, and we observed varying degrees of NLO interference on many CDC method plates. In contrast, Legiolert was resistant to NLO interference and produced a very low rate of false-positive results. Collectively, Legiolert is a sensitive and specific method for quantification of *L. pneumophila* from non-potable water that provides advantages over the CDC method.

## Introduction

Legionnaires’ disease is a significant world health problem that is increasing in frequency [1, 7, 26]. Forty years ago, the causative agent of Legionnaires’ disease was first identified as a bacterium during an investigation of a pneumonia outbreak at an American Legion convention in Philadelphia, Pennsylvania and subsequently named *Legionella* [12, 24]. The disease is now recognized as the most common waterborne disease in the United States [6]. Legionnaires’ disease is caused by inhalation or aspiration of aerosolized water contaminated with *Legionella*, which results in a severe pneumonia in susceptible individuals [10]. The disease may be nosocomial or community acquired, and risk factors include age, history of smoking, male gender, and immunodeficiency [10, 26]. Although the *Legionella* genus contains many different species, Legionnaires’ disease is predominantly caused by *L. pneumophila* [1, 7, 10, 36].

*Legionella* can be isolated from many natural aquatic environments and may also be found in soil [11, 21]. Introduction into manmade water systems allows *Legionella* to proliferate and increases the potential for infection and disease [5, 10]. Susceptible systems may include building water distribution networks, cooling towers, whirlpool spas, and industrial equipment, among others [10, 25]. Cooling towers are important due to their potential to release large quantities of *Legionella* into the air, particularly in urban environments or in proximity to susceptible individuals [18, 34]. Cooling towers have been identified as the source of infection in major fatal outbreaks occurring recently in New York City, USA [35], Québec City, Canada [31], and Warstein, Germany [23].

Proper water system management under a safety plan is important for maintaining *Legionella* concentrations below hazardous levels. Guidance on plan development and assessment of *Legionella* risk is available from multiple sources, including ASHRAE [3], the U.S. Centers for Disease Control and Prevention (CDC) [33], the American Industrial Hygiene Association [19], the U.K. Health and Safety Executive [15], the European Guidelines Working Group [8], and the World Health Organization [4]. A valuable component of a water safety plan is routine environmental monitoring for *Legionella* to ensure that implemented control measures are effective. In response to the outbreak in South Bronx, the New York State Department of Health enacted regulations requiring management of cooling towers under a plan that includes routine culture testing for *Legionella*, and which specifies corrective actions based on the level of contamination measured in the system [27]. Similarly, regulations on permissible levels of *Legionella* in cooling towers have been recently implemented in Germany [9]. In Quebec, regulations governing management of cooling towers have been in effect since 2014 and prescribe responsive actions depending on the concentration of *L. pneumophila* [14].

Traditional culture methods isolate and quantify *Legionella* species on buffered charcoal yeast extract (BCYE) media that may be supplemented with different combinations of selective agents [2, 17, 30, 32]. Because *Legionella* is relatively slow growing, other heterotrophic bacteria in the water sample can interfere with the test, for example by competing for nutrients or producing inhibitory secondary metabolites [13, 20]. Culture methods, therefore, typically employ pretreatment steps utilizing acid or heat to reduce the viability of other microbial flora in the sample, as *Legionella* tend to be more resistant to these stresses [22, 30]. Such pretreatments are particularly important for non-potable waters that may contain high concentrations of interfering organisms.

Legiolert® is a new culture method for quantification of *L. pneumophila* that is based on a most probable number (MPN) approach for quantification. The test differs significantly from traditional culture methods by providing results at 7 days, rapid sample preparation and analysis, and objective interpretation of test results. A comparison of Legiolert with the ISO 11731-2 membrane filtration method for potable water [16] was recently reported [29]. In that study, Legiolert showed a high sensitivity and specificity for *L. pneumophila* and was concluded to significantly improve and simplify the detection of *L. pneumophila* from drinking water-related samples. In another recent study, Legiolert was compared with *Standard Methods for the Examination of Water and Wastewater* 9260J and found to provide increased sensitivity for *L. pneumophila* in potable water, and equivalent sensitivity for detection from non-potable water [28]. In the present study, we compared the performance of Legiolert with the CDC method [32] for detection of *L. pneumophila* from non-potable samples. A split-sample analysis was conducted in which a population of samples, primarily from cooling towers, was tested in parallel with each method. Collectively, we found Legiolert to be a sensitive and specific method for quantification of *L. pneumophila* from non-potable water that provides advantages over the CDC method.

## Materials and Methods

### Samples

A total of 288 non-potable water samples were analyzed, comprising those submitted to our laboratory for routine testing between June and September 2016. Samples were predominantly from cooling systems including cooling towers and evaporative condensers (238). A smaller number of samples were from other industrial water systems (13), decorative fountains (2), and hot tubs (2). For the remaining non-potable samples, a specific source was not indicated (33). Geographically, most samples originated from Texas (205), with the rest coming from 6 States in the Midwest (32) and 7 in the Southeast (25). Location information was not present for 26 of the non-potable samples.

### CDC Method

Samples were plated on different agar formulations derived from BCYE (HealthLink-Clorox Healthcare, Jacksonville, U.S.). PCV medium was supplemented with polymyxin B (100 units mL^−1^), cycloheximide (80 µg mL^−1^), and vancomycin (5 µg mL^−1^). GPCV medium was prepared identical to PCV except that it was also supplemented with glycine (0.3% m v^−1^). For each water sample tested, 0.1 mL was direct plated on one BCYE plate, and two plates each of PCV and GPCV. A portion of each sample was acid treated by mixing 1.0 mL of sample with 1.0 mL of KCl-HCl acid buffer and incubating for 15 min at room temperature. Acid buffer was prepared by combining 18 parts 0.2M KCl and 1 part 0.2M HCl. A 0.1 mL aliquot of acid-treated sample was then plated on one BCYE plate, and two plates each of PCV and GPCV. Agar plates were incubated at 35 ± 2 °C with humidity and increased CO_2_ for up to 7 days. Plates were examined for *Legionella* growth after 72 to 96 h of incubation, and again after 7 days. Each distinct colony displaying presumptive *Legionella* morphology was counted, and one representative of each type was confirmed by streaking to both BCYE and BCYE without cysteine (BCYE-Cys) plates. After incubation of the confirmation plates for 2–4 days at 35 ± 2 °C, isolates that formed colonies on BCYE but failed to grow on BCYE-Cys were considered presumptive *Legionella*. Growth on confirmation plates was examined under UV illumination and fluorescent isolates were regarded as non-pneumophila *Legionella* species. In about half of cases, the species and serotype of *Legionella* was confirmed by direct fluorescent-antibody (DFA; m-Tech Monoclonal Technologies, Inc., Milton, U.S.) or latex agglutination (*Legionella* Latex Test Kit; Oxoid, Basingstoke, U.K.). Non-fluorescent presumptive colonies were occasionally found that did not react with the DFA or latex reagents; these *Legionella*-like isolates were considered non-pneumophila *Legionella* species for the purposes of this study. The number of interfering, non-*Legionella* colonies on each plate was estimated and assigned to one of the following six categories: none, 1–5, 6–25, 26–100, 101–300, or >300.

### Legiolert

The Legiolert test detects *L. pneumophila* using a substrate present in the Legiolert reagent that leads to production of a brown color indicator. For non-potable water, the limit of detection is one organism per 0.1 mL of sample, and the test employs a pretreatment step to reduce interference from non-*Legionella* organisms. Testing was performed per the manufacturer’s instructions (IDEXX Laboratories, Inc., Westbrook, U.S.). For each test, 100 mL of sterile deionized water was aliquoted into a 120 mL polystyrene vessel. The contents of one Legiolert blister pack was added and the vessel shaken to dissolve the reagent powder. Pretreatment of each sample was performed by mixing 0.2 mL of sample with 0.2 mL of prepared Pretreatment solution in a 1.5 mL microtube. After 60 s of incubation at room temperature, a 0.2 mL aliquot of treated sample was promptly transferred into the vessel containing dissolved Legiolert. Vessel contents were mixed, poured into a Legiolert Quanti-Tray, and sealed with a Sealer PLUS (IDEXX Laboratories, Inc., Westbrook, U.S.). Trays were incubated at 37 ± 0.5 °C with humidity. After 7 days, positive wells were identified by the presence of brown color and/or turbidity when compared to an uninoculated, negative-control tray. Positive wells in Legiolert typically show brown color, however, in some cases a weak color reaction may occur where the well does not appear brown but does show turbidity. Positive wells in Legiolert are, therefore, indicated by either brown color and/or turbidity. At least 25% of the positive wells in each tray were randomly tested to determine whether the observed reaction was due to *L. pneumophila*. Testing was performed by streaking approximately 5 µL of culture extracted from a positive well on both BCYE and BCYE-Cys plates. Streaks showing isolated colonies that grew on BCYE but failed to grow on BCYE-Cys were regarded as presumptive *Legionella*. In most cases, when serotyping was performed on isolates from the CDC method, it was also performed on Legiolert isolates from the same sample.

### Data Analysis

Sensitivity and specificity calculations were performed as follows. Sensitivity was defined as the probability of obtaining a true positive result from a known positive sample. Specificity was defined as the probability of obtaining a true negative result from a known negative sample. A sample was considered a known positive if a confirmed *Legionella* was identified from the sample in this study by either Legiolert or the CDC method. A sample was considered a known negative if both methods failed to detect *Legionella* from that sample. Separate sensitivity and specificity calculations were performed to examine both *L. pneumophila* and *Legionella* species detection independently. For example, to calculate the sensitivity of Legiolert for *L. pneumophila*, the total number of samples in which *L. pneumophila* was detected by Legiolert (76) was divided by the total number of samples in which *L. pneumophila* was detected by either Legiolert or the CDC method (91), giving a probability of 0.84. To calculate the specificity of Legiolert for *L. pneumophila*, the total number of samples that were correctly identified as negative by not producing a true- or false-positive result (191) was divided by the total number of samples in which *L. pneumophila* was not detected by either Legiolert or the CDC method (197), giving a probability of 0.97. Results from two samples showed a mixture of true- and false-positive wells in the same tray, however, the majority of positive wells in each case were found to contain *L. pneumophila* (75 or 89%, respectively) and these samples were considered true positives in the calculations. Sensitivity and specificity for the CDC method were calculated similarly; however, because the CDC method includes a confirmation step as part of the method, no false-positive results were produced and the resulting specificity was 1.00. Confidence intervals for each population proportion were calculated at the 95% level using z* = 1.96.

To compare Legiolert and the CDC method for specific, quantitative detection of *L. pneumophila*, the data were filtered and normalized as follows. First, results corresponding to non-pneumophila *Legionella* species were excluded. For samples in which *L. pneumophila* was found along with a non-pneumophila *Legionella* species, only the *L. pneumophila* result was retained. Second, results were excluded if a valid count was not obtained for one of the methods, for example, due to unreadable agar plates or a tray showing all wells positive. Third, Legiolert results were excluded when confirmations indicated a false-positive result. In the two cases described above where Legiolert wells containing *L. pneumophila* were found in the same tray with an infrequent false-positive well, the fraction of true positives determined by confirmation was assumed to represent the fraction of true positives in the entire tray and the MPN was prorated to reflect only the *L. pneumophila* result. Finally, results for the CDC method were expressed on a consistent basis of CFU per 0.1 mL by averaging counts from replicate plates (PCV or GPCV), and doubling results from acid-treated conditions to account for the additional dilution. The highest *L. pneumophila* result obtained from among the six different CDC plating conditions was compared with the Legiolert *L. pneumophila* result for that same sample. Differential performance of the methods was evaluated using a two-tailed Wilcoxon signed-rank test for paired continuous data, and McNemar’s test for paired categorical data. The signed-rank test was used because the distribution of differences between the methods was non-normal (Shapiro–Wilk test, *P* < 0.0001). Statistical analyses were performed in JMP software, version 13.0.0 (SAS Institute Inc., Cary, U.S.).

## Results

Of the 288 non-potable water samples that were analyzed, 96 (33%) were confirmed positive for *Legionella* by either the CDC or Legiolert methods (Table 1). Examination of isolates from both methods with UV illumination and serotype testing showed that 91 of these contained *L. pneumophila*, and 10 contained a non-pneumophila *Legionella* species. The non-pneumophila *Legionella* species were isolated exclusively using the CDC method. In all cases, *Legionella* isolates purified from positive Legiolert wells were found to be *L. pneumophila*, and serotyping showed these to be predominantly type 1, with examples of types 4, 5, and 6 also found. This matched the pattern of serotypes isolated from the CDC method, and in all 27 cases where serotyping was performed on *L. pneumophila* found by both methods, the serotypes isolated from each method were identical.

**Table 1:** Frequency of *L. pneumophila* and non-pneumophila *Legionella* species detection from non-potable water by Legiolert and the CDC method

*Legionella* species	Number of positive samples		
	Legiolert	CDC	Both methods
*L. pneumophila*	76	74	
Non-pneumophila *Legionella*	0	6	
*L. pneumophila* and non-pneumophila (mixture)	0	4	
Total: *L. pneumophila*	76	78	91
Total: all *Legionella* species	76	84	96

A comparison of the sensitivity and specificity shown by Legiolert and the CDC method was conducted on a per-sample basis. Two sets of calculations were performed to examine results for detection of *L. pneumophila* separately from results with all *Legionella* species included, as public health guidelines exist with action limits for each parameter. As shown in Table 2, Legiolert and the CDC method showed very similar sensitivity for *L. pneumophila* at 0.84 ± 0.08 and 0.86 ± 0.07, respectively. The two methods also showed high specificity. When results for all *Legionella* species were examined, a higher level of sensitivity was observed for the CDC method due to the detection of non-pneumophila *Legionella* species that, as expected, were not found with Legiolert due to its specificity for *L. pneumophila*.

**Table 2:** Sensitivity and specificity calculations

Species	Method	Sensitivity^a^	Specificity^a^
*L. pneumophila*	Legiolert	76/91 = 0.84 ± 0.08	191/197 = 0.97 ± 0.02
	CDC	78/91 = 0.86 ± 0.07	197/197 = 1.00
All *Legionella* species	Legiolert	76/96 = 0.79 ± 0.08	186/192 = 0.97 ± 0.02
	CDC	84/96 = 0.88 ± 0.07	192/192 = 1.00

^a^Sensitivity: the number of true-positive samples found was divided by the total number of known positive samples (all methods). Specificity: the number of true-negative samples found was divided by the total number of known negative samples (all methods). Calculation methodology is described in more detail in the “Materials and Methods”

The performance of Legiolert and the CDC method for specific quantification of *L. pneumophila* was examined. For quantitative analysis, the data were first processed as described in the “Materials and Methods”, and results for two samples containing *L. pneumophila* were excluded because a valid count was not obtained for both methods. In the first case, all the CDC method plates were unreadable due to extensive growth of interfering non-*Legionella* organisms. In the second case, the Legiolert tray showed all wells positive, and although this tray was shown to contain *L. pneumophila*, the test provided only a lower boundary for the resulting MPN in this instance (> 2272.6 per 0.1 mL) that could not be compared quantitatively to the CDC result. After exclusion of these results and those corresponding to non-pneumophila *Legionella* species, data from 89 samples were available for quantitative comparison.

**Fig. 1:**
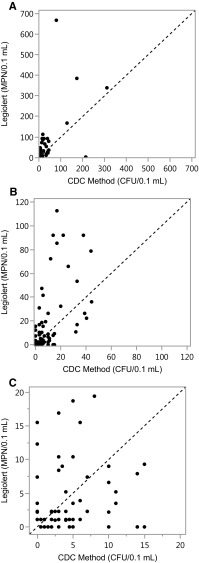
Bivariate plots comparing *L. pneumophila* detection by Legiolert and the CDC method. Each plot, “A”, “B”, and “C”, shows the same data with different axis scaling. The dotted line represents equivalent performance between the methods

A bivariate plot of the method comparison is shown in Fig. 1. The MPN for *L. pneumophila* in Legiolert ranged from 0 to 667.6 with a mean of 32.0 and a median of 3.5. Counts of *L. pneumophila* from the CDC method ranged from 0 to 311, with a mean of 18.3 and a median of 5.0. Results at the upper ends of these ranges were uncommon and most samples (83/89) were found by both methods to be below 100 MPN or CFU, with a majority of these (65/89) yielding estimates in the range from 1 to 20 MPN or CFU. Matched-pair analysis using the Wilcoxon signed-rank test indicated Legiolert and the CDC method were not significantly different for quantification of *L. pneumophila* (Table 3; *P* = 0.188).

**Table 3:** Comparison of *L. pneumophila* quantification by Legiolert and the CDC method

Group	Group criteria^ab^	Number of samples	Group mean^a^		Group median^a^		*P* value^c^
			Legiolert (MPN)	CDC (CFU)	Legiolert (MPN)	CDC (CFU)	
1	None (all data)	89	32.0	18.3	3.5	5.0	0.188
2a	<20	67	4.6	4.1	1.1	3.0	0.482
2b	>20	22	115.4	61.3	69.1	33.0	0.003
3a	<10	60	3.0	3.6	1.1	3.0	0.110
3b	>10	29	92.0	48.6	41.6	22.0	0.001
4a	<30	72	6.3	5.2	2.2	3.3	0.671
4b	>30	17	140.6	73.6	85.4	39.0	0.007

^a^Values expressed as MPN or CFU per 0.1 mL of sample

^b^Samples were assigned to “a” or “b” subdivisions within each group based on the mean of the Legiolert and CDC result for each sample

^c^Two-tailed *P* value from matched-pair analysis of each group with the Wilcoxon signed rank test

Closer inspection of the results suggested Legiolert may be more sensitive with a subset of samples containing higher concentrations of *L. pneumophila*. This trend is apparent from Fig. 1 and reflected in the differences between the group means and medians for each test. To investigate this possibility, the matched-pairs analysis was repeated after dividing the data into two groups according to the mean of the Legiolert and CDC result for each sample. When the 67 samples giving mean results below 20 MPN or CFU were examined (Table 3: Group 2a), matched-pair analysis showed no significant difference (*P* = 0.482) and both methods provided similar overall estimates of the *L. pneumophila* concentration for all samples in the group. Conversely, a significant difference was observed with the reciprocal group of 22 samples having mean results above 20 MPN or CFU (*P* = 0.003). This difference may be due to the broader counting range of Legiolert (1-2273 MPN) compared to an agar plate (typically 1-300 CFU), or the resistance of the MPN approach to the negative effects of dense colony growth that can occur with agar plates due to the compartmentalization of sample in the Legiolert Quanti-Tray device. This finding was not dependent on a specific point of division between the two groups, and similar results were also obtained when the data were partitioned at 10 or 30 MPN or CFU (Table 3).

A further quantitative analysis was performed to determine whether Legiolert and the CDC method differed in the proportions of samples found positive for *L. pneumophila*. Collectively, both methods gave 259 concordant results and 27 discordant results (Table 4). McNemar’s test indicated the division of discordant results between Legiolert and the CDC method was not significant (*P* = 0.700). In 21 of 27 cases, the positive sample in each pair showed a low magnitude count in the range from 1 to 5 MPN or CFU; these results were close to the limit of detection and would be expected to show inconsistent detection. In the remaining 6 cases, the discordant positive result ranged from 7.4 to 15.5 MPN or CFU and these were evenly distributed between the two methods.

**Table 4:** Presence or absence of *L. pneumophila* in cooling tower samples according to Legiolert or the CDC method

Legiolert result	CDC result		Row total
	Positive	Negative	
Positive	62	12^a^	74
Negative	15^a^	197	212
Column total	77	209	286

^a^McNemar’s test shows no significant difference between these discordant results (*P* = 0.700)

Non-*Legionella* organisms (NLO) were found on many of the agar plates used for the CDC method (Table 5). When such growth was extensive or confluent, plates were unreadable and detection of *Legionella* was precluded. PCV and GVPC provided the highest *L. pneumophila* count for 53 (65%) of the 81 samples giving a positive result for the CDC method. In contrast, Legiolert was highly resistant to NLO interference. In most cases, no discernable impact of NLO on Legiolert was observed when samples showing significant NLO growth on CDC plates were tested. In one notable example, Legiolert detected a significant concentration of *L. pneumophila* (98.9 MPN per 0.1 mL) from a sample that was judged unreadable on all six CDC plating conditions. Nevertheless, Legiolert was affected by NLO in a small number of cases, and false-positive results were observed in 6 samples where *L. pneumophila* was not detected. Results for two other samples showed a mixture of positive wells with most found to be true positives containing *L. pneumophila* (75 or 89%, respectively) and the rest found to be false positives. Collectively, confirmation testing across all samples isolated only NLO from 14 (4.9%) of 286 randomly tested positive wells. Because *L. pneumophila* was isolated together with a NLO from true-positive wells in some cases (<10%), the presence of NLO alone is insufficient to determine true or false positivity; however, the 14 wells in question also exhibited atypical appearance or coloration and were thus concluded to be false positives for the purposes of this study. In all cases, the water samples from which these false-positive Legiolert wells were observed also showed significant NLO interference on the CDC method plates. Among this group of samples showing false-positive wells, *L. pneumophila* was identified by both methods in three cases, and none of the samples were found to contain non-pneumophila *Legionella* species.

**Table 5:** Frequency of non-*Legionella* organisms (NLO) observed on CDC method plates

Degree of NLO impact	Media formulation and pretreatment					
	BCYE (%)	PCV (%)	GPCV (%)	BCYE acid (%)	PCV acid (%)	GPCV acid (%)
None^a^	4	10	21	14	45	64
Potential for interference^b^	21	54	58	50	53	35
Unreadable^c^	74	35	21	35	1	0

^a^NLO were not observed

^b^Countable colonies of NLO were observed at one of the following levels per plate: 1–5, 6–25, 26–100, 101–300

^c^Plates judged unreadable when >300 colonies of NLO were observed

## Discussion

Non-*Legionella* organisms present a significant challenge for enumeration of *Legionella* from non-potable samples. To suppress these interfering organisms, pretreatment with acid or heat is typically used in combination with selective media. In this study, only acid pretreatment was used according to the CDC protocol [32], however, it is possible that inclusion of a heat pretreatment step may have improved detection of *Legionella* in some samples. Because these approaches can also affect *Legionella* detection, a variety of plating conditions are traditionally used to maximize recovery by providing only as much selectivity as is necessary. The result is a complex method that requires significant time, resources, and expertise to perform. The primary benefit of a traditional culture method is the ability to detect different *Legionella* species. This makes it well-suited to epidemiological case investigations, where the need for diverse species detection and identification justifies the added complexity and necessity for specialized laboratory expertise. Although *L. pneumophila* cells from a positive Legiolert well can be removed for immediate serotyping, storage, or other testing, an additional benefit to a traditional culture method is that an experienced analyst can use colony morphology to select particular isolates for follow-up testing.

This evaluation showed Legiolert provides advantages over traditional culture methods that make it better suited for routine monitoring applications. First, Legiolert employs a simple sample preparation and test procedure that can improve laboratory workflow and efficiency. Second, positive wells were easy to identify and could be counted rapidly, accurately, and with little to no interpretation. This differs from plate-based methods where the various colony morphologies presented by *Legionella* and NLO require analyst judgement and selection of appropriate candidates for confirmation. Third, Legiolert was robust to NLO interference and produced only a small number of false-positive results. This high selectivity allowed samples not containing detectable *L. pneumophila* to be rapidly processed due to the absence of any positive signal. In contrast, many of the CDC method plates for these same negative samples required significant analyst time and effort due to the frequent presence of NLO that necessitated examination and judgement. Finally, and arguably most importantly, Legiolert showed similar overall sensitivity for *L. pneumophila* when compared to the CDC method, but demonstrated increased sensitivity with samples containing higher *L. pneumophila* concentrations. The latter result is noteworthy because it indicates that the CDC method may tend to underestimate the concentration of *L. pneumophila* in samples that are more highly contaminated. In these cases, Legiolert appears more likely to give a result leading to the appropriate corrective action corresponding to the true concentration of *L. pneumophila* in the system. Collectively, the results of this study show that Legiolert provides an effective and efficient method for detection of *L. pneumophila*, the primary pathogenic *Legionella* species. The combined benefits to performance and usability make Legiolert particularly advantageous for routine monitoring of non-potable water systems.
